# Azimuthal sound localization in the chicken

**DOI:** 10.1371/journal.pone.0277190

**Published:** 2022-11-22

**Authors:** Gianmarco Maldarelli, Uwe Firzlaff, Harald Luksch

**Affiliations:** Chair of Zoology, School of Life Sciences, Technical University of Munich, Freising-Weihenstephan, Germany; University of Alberta, CANADA

## Abstract

Sound localization is crucial for the survival and reproduction of animals, including non-auditory specialist animals such as the majority of avian species. The chicken (*Gallus gallus*) is a well-suited representative of a non-auditory specialist bird and several aspects of its auditory system have been well studied in the last decades. We conducted a behavioral experiment where 3 roosters performed a sound localization task with broad-band noise, using a 2-alternative forced choice paradigm. We determined the minimum audible angle (MAA) as measure for localization acuity. In general, our results compare to previous MAA measurements with hens in Go/NoGo tasks. The chicken has high localization acuity compared to other auditory generalist bird species tested so far. We found that chickens were better at localizing broadband noise with long duration (1 s; MAA = 16°) compared to brief duration (0.1 s; MAA = 26°). Moreover, the interaural difference in time of arrival and level (ITD and ILD, respectively) at these MAAs are comparable to what measured in other non-auditory specialist bird species, indicating that they might be sufficiently broad to be informative for azimuthal sound localization.

## Introduction

Sound localization is crucial for the survival and reproduction of animals—e.g., to avoid predators, catch prey and detect possible mates. Numerous experiments have been conducted in the last few decades in order to understand how birds locate sounds [1]. The avian species studied best in this regard is the barn owl (*Tyto alba*), a nocturnal bird of prey with remarkable abilities to hunt in total darkness by using sounds as the only cue [2, 3]. The sound localization ability of barn owls has been measured in controlled conditions through behavioral experiments, showing outstanding performances [4–8]. Several experimental paradigms have been developed to measure the behavioral response to change in sound location, such as tracking saccadic head movements toward the target stimulus with the search coil technique [4, 5], measuring the pupillary dilation response [6, 7], and behavioral training in a Go/NoGo task [8]. Besides these experiments, other studies described the anatomical specializations which provide high auditory localization acuity (i.e., asymmetrical ears and facial ruff; [4, 9–11]), as well as the neuroanatomy and the physiological mechanisms underlying the computation and representation of auditory space in the brainstem (for a review: [12, 13]). Consequently, some studies could accurately predict and explain aspects of the sound localization behavior of the barn owl according to the computation of the underlying neural substrates [14, 15].

However, most avian species—referred to here as generalist birds—have symmetrical ears and lack any auditory specialization for improving sound localization. To our knowledge, only a few studies measured the sound localization accuracy in auditory generalist species [16–20]. Within this group, the chicken (*Gallus gallus*) is a well suited representative of this avian group, since several aspects of its auditory system—such as the anatomy, physiology, evolution and development—have already been studied (for reviews: [21, 22]). Moreover, it has already been used as a model for the plesiomorphic condition of the avian auditory system and compared to the auditory-specialist barn owl [13, 23]. Several studies were conducted in the chicken’s brainstem nuclei, showing, for instance, the neural processing of binaural cues, fundamental for sound source localization [24, 25], and the role of the interaural canal in enhancing the interaural time difference (ITD) range [26, 27]. In a study from our own lab, we could demonstrate the presence of head-induced cues that might be used by the auditory system to improve the localization acuity, especially in elevation [28]. Apart from a recent paper which shows that hens are able to locate sound sources along azimuth with high accuracy [29], little is known about the psychophysics of the chicken’s sound localization. We conducted a behavioral experiment where roosters performed a sound localization task while broad-band noise was presented from different loudspeakers along azimuth. We quantified the localization acuity by calculating the minimum audible angle (MAA) [30]. We show that chicken’s performance is remarkably good among generalist bird species and discuss the factors contributing to this result.

## Materials and methods

### Animals

In total the behavioral training involved 15 chickens (*Gallus gallus*, White Leghorn; 13 males, 2 females). However, in our first behavioral cohort the female subjects did not learn the final task (see ‘Training’ section), so we continued to train males and eventually collected data for the final task from 3 male chickens aged between 100 and 160 days post-hatch. Fertilized eggs were provided by the Chair of Reproductive Biology, TUM School of Life Sciences, incubated at 37°C and 70% humidity and, after hatching, reared at the animal facility of the Chair of Zoology, TUM School of Life Sciences. The animals were kept in groups in cages with access to sand, perches, water, and food *ad libitum*. The bird housing facilities were artificially illuminated with UV-balanced light in a 12 h/12 h light-dark cycle. After the period where the animals were tested for this study, they were used in electrophysiological experiments. At the end of those experiments the animals, which were kept anaesthetized by a constant injection of anesthetic (ketamine: 13mg/kg/h; xylazine: 4mg/kg/h), were sacrificed with an intrapulmonary injection of sodium-pentobarbital (200mg/kg, Narcoren) and decapitated with poultry scissors. All experiments were performed according to the principles regarding the care and use of animals adopted by the German Animal Welfare Law for the prevention of cruelty to animals. This study (including the mentioned electrophysiological experiments) was approved by the Government of Upper Bavaria, Germany (permit no. ROB-55-2-2532-Vet_02-18-154).

### Stimuli presentation

The stimuli were broadband (BB) noise (0.4–4 kHz), in the frequency range corresponding to the highest chicken’s sensitivity, according to its audiogram [31]. A ramp function of 10 ms was applied at the beginning and the end of the stimuli; the time duration was 0.1 s or 1 s, depending on the experimental paradigm. In this study, the experimental condition with presentation of BB noise with 1 s duration is called “closed-loop condition”, whereas the presentation of BB noise with 0.1 s duration is called “open-loop condition”. In the literature the terms “closed-loop” and “open-loop” typically refer to the presence or absence of an orienting head movement used to enhance sound localization, as observed, e.g., in the barn owl [5]. In our study we use these terms because, regarding the long duration sounds, an active head movement was observed (see S1 File), whereas, for the short duration stimuli, a duration of 100 ms would not be long enough to allow the chicken to respond with a head movement. The barn owl, for example, which is an auditory specialist, has a head turn latency of about 100 ms [5, 32]. Thus, the localization accuracy in this case would solely depend on the ‘snapshot’ binaural cues. The stimuli were generated by a custom-written script in MATLAB (MathWorks, USA), converted to analogue signals via an external sound card (Fireface 400, RME, Germany), amplified (AX-396, Yamaha, Japan) and presented through the loudspeakers (Cougar NSW1-205-8A, AuraSound, USA).

11 loudspeakers were mounted on a semi-circular aluminum structure (radius = 50 cm; from now on called ‘loudspeakers hoop’) mounted at the chamber wall opposite from the entrance. The center of the loudspeaker hoop semi-circle was at the position where the head of the chicken was supposed to be during the stimuli presentation. The loudspeakers were covered with an acoustically transparent but optically opaque cloth to prevent visual cues from membrane movement. Each loudspeaker had been calibrated to 60 dB SPL RMS using a measuring amplifier (type 2636, Brüel & Kjaer, Denmark) and a microphone positioned at the center of the loudspeaker hoop semicircle. In order to cancel out differences between loudspeakers characteristics which could be used by the bird as a discrimination cue, we compensated the loudspeakers to obtain a flat frequency response for each of them.

### Behavioral setup

**Fig 1:**
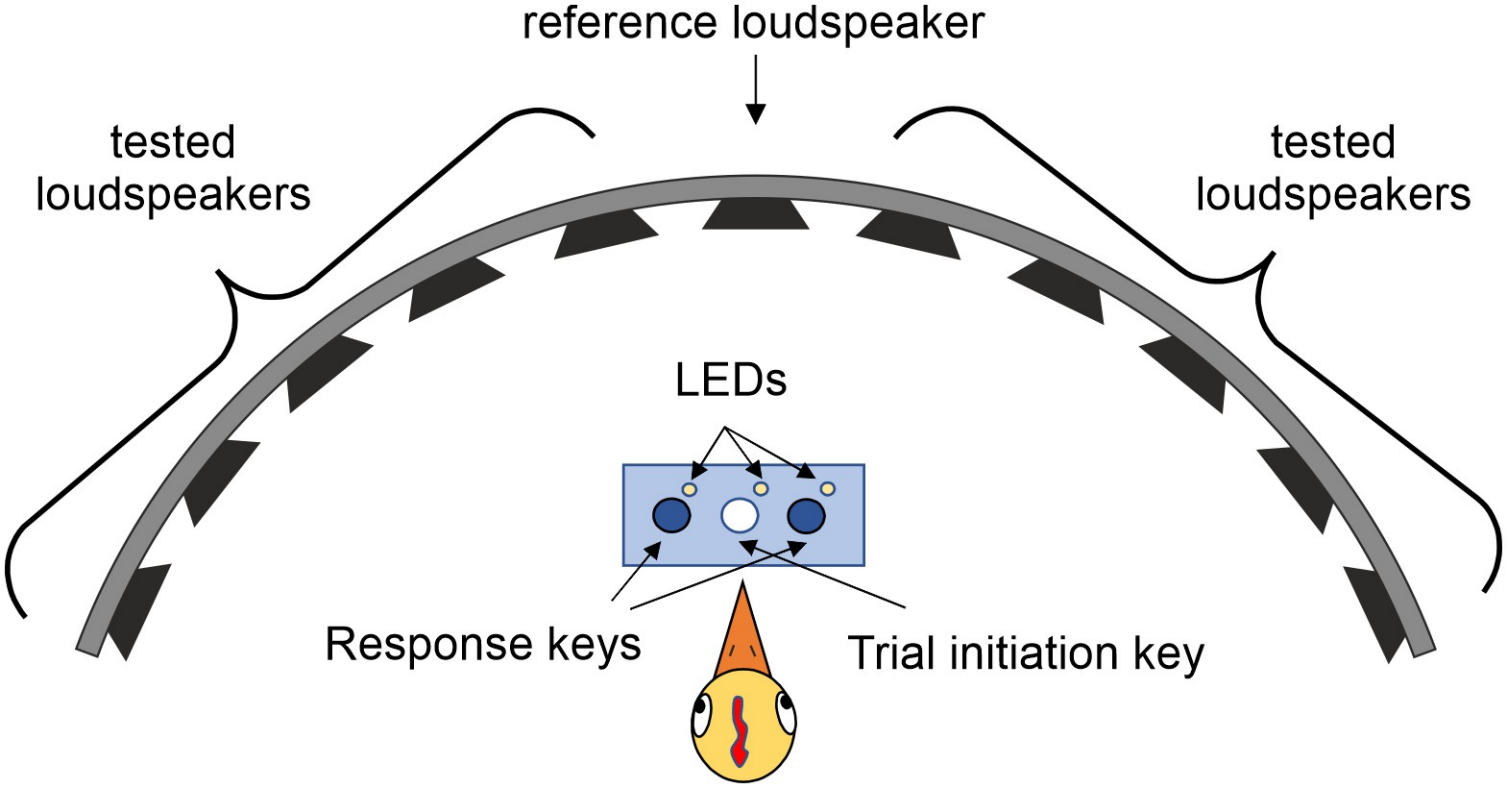
Sketch of the experimental setup.

The behavioral experiments took place in a chamber (width: 1.5 m W, 1.5 m D x 1.5 m H), coated with a 10-cm thick layer of pyramid-shaped sound-absorbing foam. The floor area where the animals could freely move was covered with a carpet to avoid sound reflections. The chamber was illuminated with two LED lightbulbs, one of which was always on, while the second one could be turned off to lower the illumination as punishment for incorrect response of the animal during training or experimental sessions (see section ‘Experimental paradigm’). A keys box was located between the loudspeaker hoop and the chicken, consisting of 3 plastic buttons, each of them placed on top of a switcher (D45U-V3LD, ZF Friedrichshafen AG, Germany). The central button was pressed by the subjects to initiate the trial, while the two lateral keys were pressed as a behavioral response to the stimuli presentation. Next to each button there was a 5-mm diameter LED (Conrad Electronic, Germany), used to provide a visual cue to the subject indicating the pre-trial period and the response period (see Section ‘Experimental paradigm’, Fig 1). A plastic-coated steel fence (1.06 m high; HEV Heimwerkermarkt GmbH & Co. KG, Germany) was mounted between the keys box and the loudspeaker hoop, in order to prevent the animals to access the loudspeakers area. In front of the chamber entrance, and behind the bird when it was facing the loudspeakers, there was a glass plate where the food pellets (Gallogold Küken Alleinkorn C, BayWa, Germany) were provided through a pipe and delivered by a custom-made food feeder placed on top of the chamber (components and assembly instructions available: www.jonasrose.net). The food plate was surrounded by an LED band (Josef Barthelme GmbH, Germany) which turned on during the food delivery, as a reward cue. A camera (30 fps, 720p; W15, Jelly Comb, USA), connected to an external Windows-operated laptop, was fixed on the door wall to monitor and record the behavior of the subjects during training and experimentation.

The control and execution of the experiments (including receiving inputs from the keys box switchers, presentation of stimuli, control of the feeder and the punishment lightbulb) was done with a custom-written MATLAB script, which allowed to control a general-purpose input/output device (K8055, Velleman, Belgium).

### Behavioral training

The behavioral training involved 15 chickens (13 males, 2 females). The choice to train mainly males was justified by preliminary training (tested in 3 males and 5 females, where 2 roosters learned the final task), in which females showed a motivational drop during the last steps of the training paradigm and could not even learn to localize the most lateral sounds (at– 65° and + 65° azimuth). The training started from the age of 5–12 days post-hatch and was divided into different tasks with increasing difficulty until reaching the final experimental task. It was based on operant conditioning and positive reinforcement. At the beginning, after familiarization with the trainers, the birds were rewarded every time they pressed a button with the beak. Then, the animals were trained to press only the buttons indicated by a visual cue, i.e., the corresponding LED. Starting from the third week post-hatch, the birds could be trained alone without social isolation distress. When the animals reached high performance at following the visual cues, the auditory cues were also included, by playing sounds at the most lateral positions. At this stage, the LED of the correct response key was turned on with a delay after the target sound presentation, creating an advantage in reward time for the animals that used the sound as a main cue. Then, both lateral LEDs were turned on, removing any information content of the visual cues. When the animal reached high performance at this task (i.e., the performance was higher than chance level according to the binomial test, p≤0.01; see below “Statistical analysis”), the target sounds at smaller angles were gradually included, until the presentation of all of them. At this point the animal could be tested in the final task.

### Experimental paradigm and data collection

The task was a relative sound localization task, i.e., the subject had to localize the direction of a target sound in relation to a reference sound, tested using a 2-alternative-forced-choice (2AFC) paradigm. The trials were grouped into sessions, each of them containing 30–48 trials. Within each session, the number of repetitions for each target sound location ranged between 3 and 4, and the order of the stimuli presentation was pseudo-randomized. Between sessions there was a 5-minute break.

At the beginning of each session, the animal was placed into the chamber, and the experiment was performed with the door closed. Each trial consisted of the following sequence of events: the subject pressed the central key for the trial initiation; a BB noise was presented from the central loudspeaker (the reference stimulus); after 0.5 s of inter-stimulus interval, the same sound was presented from one of the lateral loudspeakers (the target stimulus); at the end of the second stimulus presentation, the subject pressed one of the lateral keys to report the direction of the second sound in relation to the first one. The response period was indicated by illumination of both lateral LEDs. The correct response was pressing the key at the same side of the second target sound. In that case, the food plate LEDs turned on for 3 s and the reward food was delivered onto the plate. In case of wrong responses (i.e., pressing the key at the opposite side of the target stimulus) the illumination was dimmed for a period of 2–3 s. In case the subject pressed the central key, the same punishment was applied. The response period had a duration of 4–5 s, therefore if the subject did not respond within that time window, a new trial could be initiated by pressing the central button.

### Data analysis

Given the high variability in the subjects’ motivation across time, we applied some criteria for the selection of the valid sessions to be included for the data analysis, divided into two steps.

During the response time window of each trial, the subject could show 2 behaviors in addition to the typical lateral responses: 1) pressing the central key or 2) not providing any response. Since a high rate of these undesired behaviors indicates a lack in motivation or inability of performing the task, as a first step we discarded the sessions where the rate of lateral pecking responses was < 75% of the total trials.

Secondly, when the animal consistently reported lateral responses, we expected the localization performance to be above chance level for wide angles between reference and target stimuli. Considering that the MAA of the starling (*Sturnus vulgaris*), another generalist bird, is equal to 17° [20], we assumed that angles ≥ 52° are sufficiently wide to be easily localized by the chicken. These angles corresponded to the two leftmost and two rightmost loudspeakers on the loudspeaker hoop (-52°, -65° and + 52°, + 65°). However, in some cases we observed a biased response strategy, where the subject responded systematically (or preferentially) to one of the two lateral keys, regardless of the target location. In order to discard these inappropriate sessions, we only considered the sessions where the proportion of correct responses to the external loudspeakers was significantly high and not due to chance (see ‘Statistical analysis’ section). The appropriate sessions were used for the calculation of the MAA.

The procedure to calculate the MAA is comparable to what has been done in a similar study on humans [30]. The proportion of the responses to the right key has been plotted as a function of the angle between reference and target stimulus. After fitting the curve to the data (see ‘Statistical analysis’ section), it was possible to calculate the angle at which the proportion of responses was equal to 0.25 and 0.75 (A_25_ and A_75_, respectively). The MAA was calculated as the mean of the absolute values of A_25_ and A_75_.

### Statistical analysis

We discarded the sessions where the subject had low performance for the most external stimuli (absolute angles ≥ 52°; see section ‘Data analysis’). To do so, for each session, given the number of lateral responses for the most external stimuli, we run a binomial test to calculate the theoretical number of correct responses that would be significantly higher than chance level (p≤ 0.01, chance level probability = 0.5). If the number of correct responses for the recorded session was below this threshold, the data from that session was not included in the MAA calculation.

For the MAA calculation, a logistic function was fitted to the data points (i.e., the proportion of the responses to the right key as a function of the angle between reference and target stimulus). The logistic function had the following equation:

$$f\left(x\right)=\frac{1}{1+e^{-k\left(x-x_{0}\right)}}$$ (1)

Where *x*_*0*_ is the curve’s midpoint and *k* is the logistic growth rate or steepness of the function. The regression curve was fitted to the data using the non-linear least squares method. All the mentioned analyses were performed using custom-written scripts in MATLAB.

## Results

### Training

**Fig 2:**
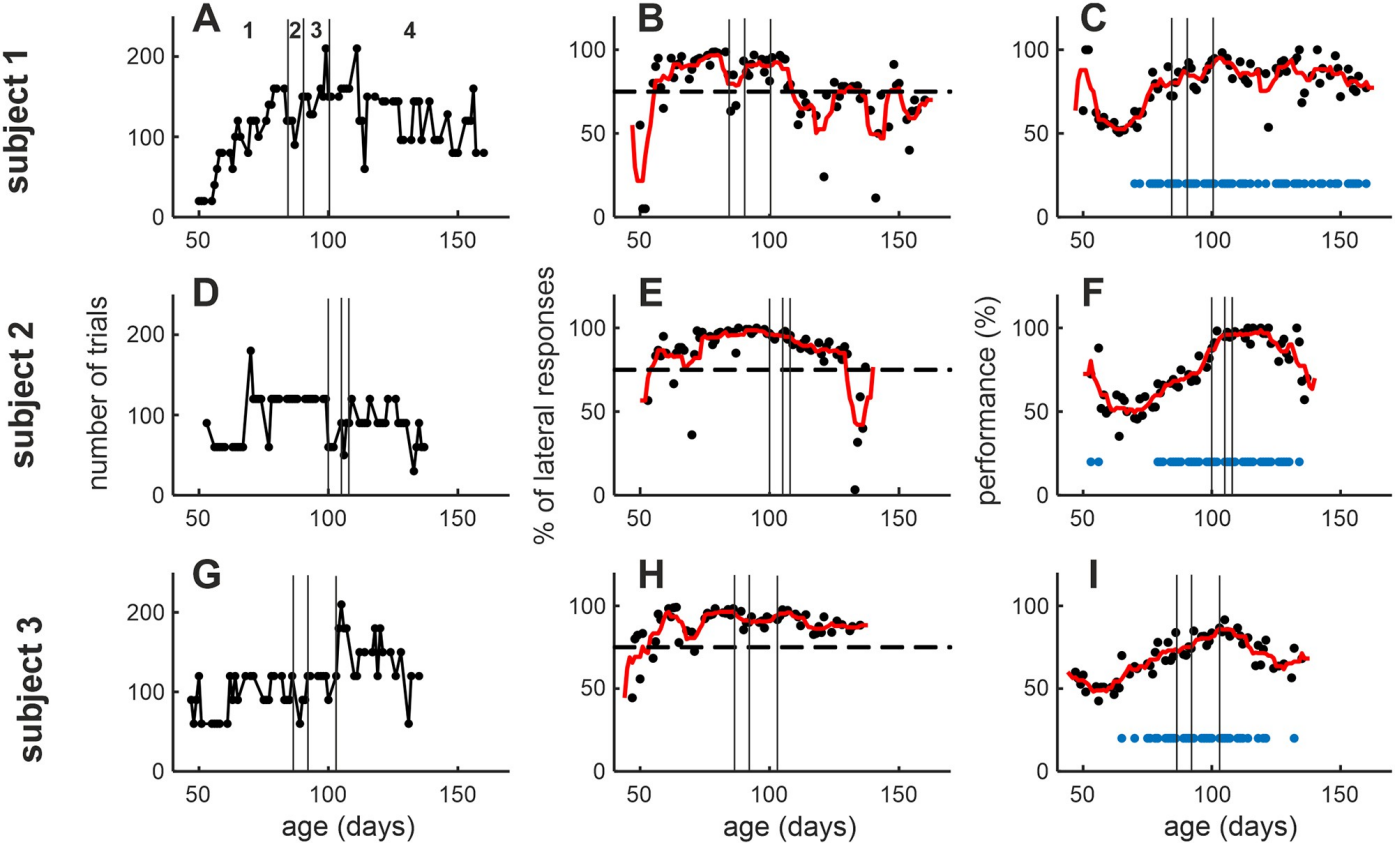
Overview of behavioral training and final task. Data about the 3 subjects which learned the sound localization task are shown. Each row refers to one subject. The time window of each plot covers a total of 4 periods between the training (from stage 1 to 3) up to the end of the experimental recording (stage 4): 1 = stimuli presentation from only external LSs, trial initiation by trainer; 2 = stimuli presentation from only external LSs, trial initiation by the subject by pressing the central key; 3 = progressive addition of stimuli with smaller angles from the reference LS; 4 = stimuli presentation from all LSs (final task). **A, D, G**) Each data point represents the daily number of trials done by the subject. Note that all 3 subjects could perform a relatively high number of trials. **B, E, H)** Each data point represents the daily proportion of lateral pecking responses over the total number of trials performed. The line is a moving mean of the data using a sliding window of 7 days. The dashed line shows the threshold used for valid session selection at 75%. **C, F, I)** The data points show the daily percentage of correct responses over the lateral key responses for the most lateral sound sources (absolute angle ≥ 52°). The line is a moving mean of the data using a sliding window of 7 days. The dots at the bottom of the plot represent the days when the performance was significantly above chance level (binomial test, p≤0.01).

For the subjects which could be tested in the final task (N = 3), the training period ranged between 91 and 100 days. Another trained subject could perform the final task, but it was not possible to conduct enough sessions for a precise estimation of the MAA. The total number of trials that the animals could perform every day was either stable over time (subjects 2 and 3) or increased up to 80 days old (subject 1) until reaching a plateau (Fig 2A, 2D and 2G). After that age, the subjects had also learned to respond by pressing the lateral keys, as the percentage of lateral responses was stably above 75%, even though subject 1 showed unstable progress (Fig 2B, 2E and 2H). The subjects were able to learn and stably perform the localization task starting from 70–80 days post-hatch. This is evident from the remarkable increase of performance in localizing sounds widely distant from the reference sound (≥ 52°), followed by a stable performance above chance level (binomial test, p≤0.01; Fig 2C, 2F and 2I). The other subjects which could not reach the final task (N = 9) were trained as long as possible, in a range between 84 and 140 days. However, they did not show any notable performance increase or sustained high-performance level. This indicates that the behavioral training had a relatively low rate of success (3/15 = 20%).

### Final experiment

**Fig 3:**
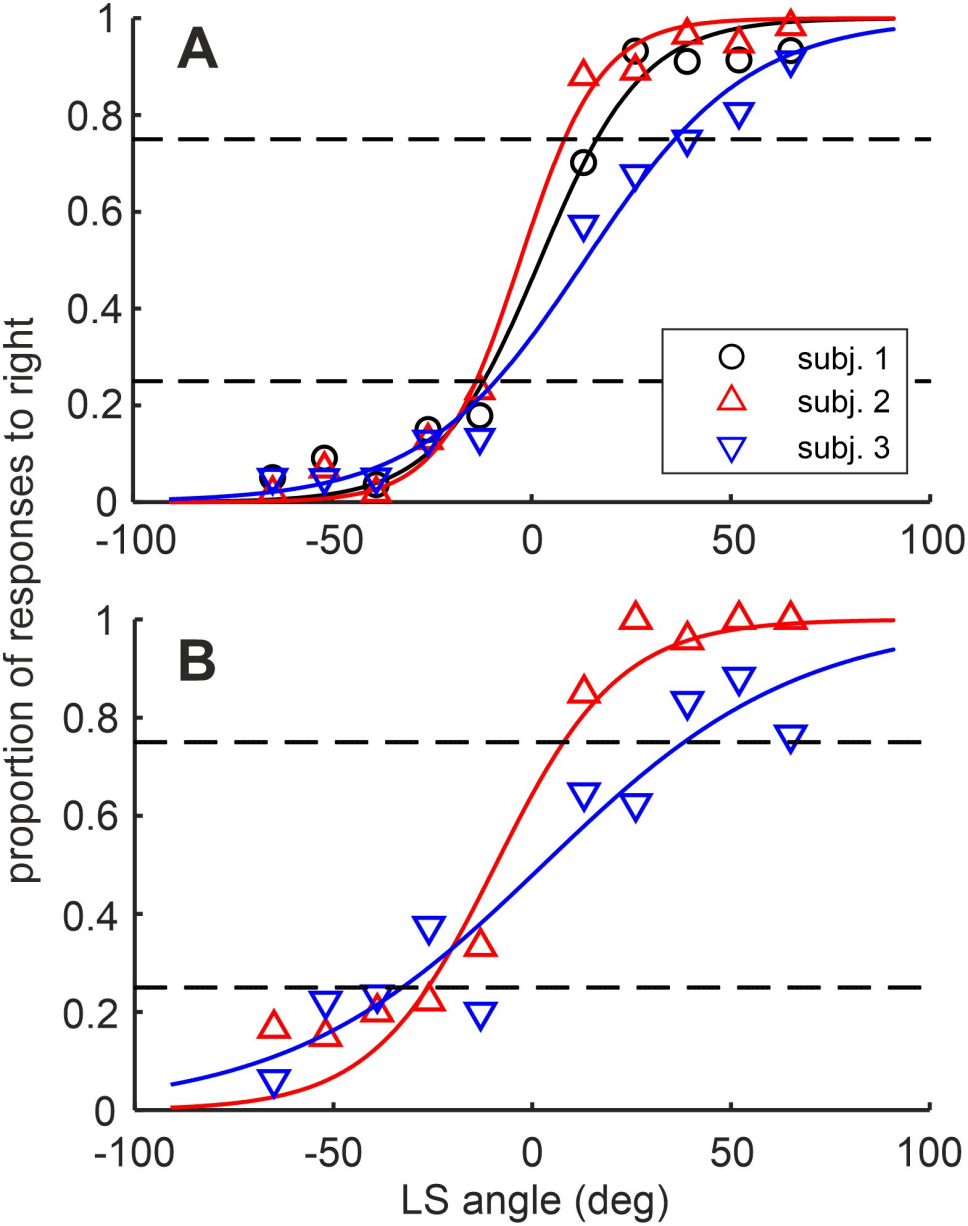
Psychometric curves of responses to the right key. Proportion of responses to the right during **(A)** closed-loop condition (stimuli duration = 1 s) and **(B)** open-loop condition (stimuli duration = 0.1 s). In both plots the lines represent the logistic curves fitted to the data of each subject. The dashed lines indicate the response proportion at 0.25 and 0.75. The MAA is calculated as the average of the absolute angles where the logistic curve is equal to 0.25 and 0.75.

The data collection period ranged between 100–108 days post-hatch and 135–160 days post-hatch (29–60 days; Fig 2). This limited time window was because the roosters, when they were around 4–5 months old, lost the motivation to perform the task (evident for subjects 2 and 3 from Fig 2F and 2I). Around that age the roosters reached sexual maturity and developed territorial and crowing behavior, the latter of which they also displayed in the experimental box. Nevertheless, all 3 subjects completed a high number of sessions and repetitions per each tested loudspeaker for the closed-loop condition (Table 1). Subjects 2 and 3 were presented also with the open-loop condition, and they could complete a lower number of repetitions per tested loudspeaker (Table 2). In general, the animals were better at localizing BB noise in the closed-loop condition compared to the open-loop one (average MAA: closed-loop = 16 ± 2°; open-loop = 26 ± 6°, Fig 3A and 3B), in line with what observed in the starling [20]. Moreover, the analysis of the head position and orientation in 3D space in one subject showed that the chickens moved the head during the closed-loop condition, and it seems to be an anticipatory movement toward the response keys (see S1 File). Conversely, the brief sounds of the open-loop condition (0.1 s) would not have allowed the animal to use the orienting head behavior to enhance the sound localization.

**Table 1:** Summary of data collection for the closed-loop condition.

Subject	Number of sessions	Number of trials	Averaged number of repetitions per sound location	R^2	MAA (°)
1	16	575	57	0.980	14
2	20	573	54	0.990	11
3	20	572	57	0.981	23

**Table 2:** Summary of data collection for the open-loop condition.

Subject	Number of sessions	Number of trials	Averaged number of repetitions per sound location	R^2	MAA (°)
1	-	-	-	-	-
2	8	211	21	0.956	17
3	6	161	16	0.895	35

### Calculation of ITD and ILD at the MAA

We calculated the ITD and ILD available at the chicken’s MAA (the “minimum audible ITD and ILD”), in order to quantify the size of these binaural cues for the azimuthal sound localization. To do so, we used 4 head-related transfer function (HRTF) datasets of adult chickens from a previous study [28]. For each HRTF sound source location, we calculated the ITDs in the tested frequency range (0.4–4 kHz) and averaged them across subjects. Then, from the linear interpolation of the averaged ITDs along azimuth, we estimated the ITDs at the positive and negative values of the MAA around the 0° azimuth. The final ITD was the average of the two estimated ITDs. The same logic was followed for the ILDs. For the closed-loop condition (mean MAA = 16°), the estimated ITD and ILD were 56 μs and 2.1 dB, respectively, whereas for the open-loop condition (mean MAA = 26°), the ITD and ILD were 92 μs and 3.1 dB. Given the ITD enhancement effect of the interaural canal that in the chicken can reach a factor of up to 1.8 [27], the ‘heard’ ITDs could broaden up to 101 μs for the closed-loop condition and 166 μs for the open-loop condition.

## Discussion

**Fig 4:**
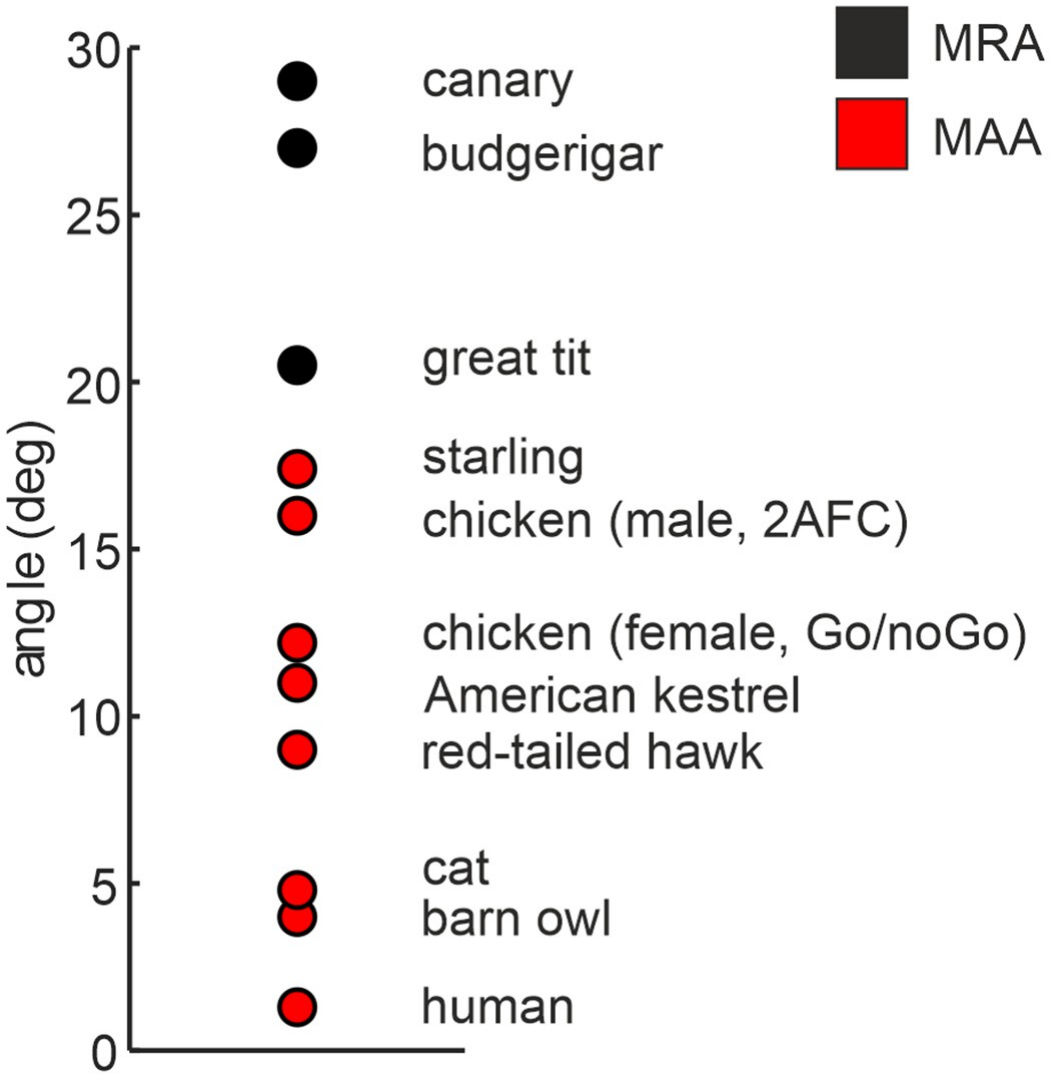
Comparison of localization acuity across species. Localization acuity measurements—minimum audible angle (MAA) or minimum resolvable angle (MRA)—in generalist birds—canary and budgerigar [19], great tit [18], starling [20], chicken, male (present study, closed-loop condition), chicken, female [29], red-tailed hawk (*Buteo jamaicensis*) and American kestrel (*Falco sparverius*) [17]–and auditory specialists such as the barn owl [8], cat [35] and human [35]. Note that the chicken shows a relatively good localization accuracy among the generalist birds, but it is much worse than avian specialists such as the barn owl or mammal species.

This study shows that freely moving roosters can localize noise along azimuth with relatively good localization acuity among the generalist bird species tested so far (Fig 4). Our data are in line with what shown for hens by Krumm and colleagues [29], where the MAA for broadband noise was 12.2°. However, in their study the hens performed a Go/NoGo task, while we used a 2AFC task. It is noteworthy that the methodological difference might affect the MAA results. The better localization acuity of chickens compared to other generalist birds might be due to the availability of wider binaural cues, which depends on factors such as the head size and the role of the interaural canal. It is known that the size and shape of the head have a relevant impact in generating the interaural differences in time of arrival and intensity (ITD and ILD, respectively) that the animal can experience at the level of the eardrums [33]. The head size of the chicken is notably bigger than most of the generalist birds shown in Fig 4 (subject 1 from the present study = 34.0 mm, starling = 15.5 mm [34], budgerigar = 15.1 mm, canary = 13.5 mm [19], great tit = 12 mm [18]) and might be comparable to the head size of the tested diurnal raptors [17]. Moreover, the chicken head shape induces monaural cues, but they are thought to be relevant for localization along elevation, rather than along azimuth [28].

The “minimum audible ITD and ILD” presented at the ear drums of the chicken for closed-loop condition are 56 μs and 2.1 dB, respectively. Budgerigars can lateralize broadband noise based on presentation of ITDs and ILDs as small as 16 μs and 1.5 dB, respectively [36], which suggests that in the chicken both binaural cues are big enough to be informative for azimuthal sound localization. For the other mentioned generalist bird species, the ITD corresponding to the MAA or minimum resolvable angle (MRA) was calculated using a model by Kuhn [37], which considers only the influence of the head size and the head shadowing in the generation of the binaural cues (within the range 1–4 kHz: budgerigar = 50–62 μs, canary = 25–55 μs [19], great tit = 18–24 μs [18], starling = 22–30 μs [20]). The minimum audible ITD of the chicken from our study is similar to the ITD calculated for these bird species. Moreover, a recent study calculated the MAA of the chicken at different pure tones, and estimated the binaural cues experienced at those MAAs [29]. The results indicate that chickens might rely on ITD cues at low frequencies and on ILD cues at high frequencies [29]. However, a combination of ITD and ILD information within the same frequency regions as in owls [38] cannot be excluded.

The ITD and ILD arriving at the ear drums are enhanced by the internally coupled middle ears of birds, which work as pressure difference receivers [39]. This effect might have a substantial influence on their localization accuracy. For instance, chicken’s interaural cavities remarkably enhance the ‘heard ITD’ by a factor of up to 1.8 [27]. Thus, in the case of the closed-loop condition, the heard ITD at the MAA might become broader up to 101 μs. Moreover, the ‘heard ILD’ is also significantly amplified in chickens [27] and might also play an important role in azimuthal localization. However, the interaural canal effect varies across bird species: for instance, the interaural canal seems to play a role in the barn owl, especially at low frequencies [40], but not in the starling [34].

Chickens were better at localizing sounds in the closed-loop condition (long stimuli duration) compared to the open-closed condition (short stimuli duration). This phenomenon has also been observed in the starling [20], but not in the barn owl [5] and the great horned owl (*Bubo virginianus*) [41]. As shown in the S1 File, in the closed-loop condition the chicken had enough time to actively move the head in order to increase the binaural cues coming to the ears, maximizing the localization acuity. In our case the head movement seems to be an anticipatory movement toward the response keys, rather than an orientation toward the sound source, as observed e.g., in the barn owl [5]. Conversely, in the open-loop condition the sound source localization should rely exclusively on the binaural cues arriving at the ear drums when the head is in the standard position, i.e., frontally oriented. It is thought that the active head orientation is the main factor responsible for the better localization performance in closed-loop paradigms [1]. However, the time duration might play a role in the localization accuracy simply due to the temporal constraints in processing brief sounds in the auditory system. Indeed, a study on humans with immobile heads showed that duration affects MAA along both azimuth and elevation [42]. However, other studies conducted on humans did not report such an effect [43, 44]. In order to disentangle the effect of motor response and auditory time integration in localization of brief sounds, future studies should be carried out e.g., where localization accuracy with freely orienting head is compared to the condition where the head is constrained in a fixed position.

Birds have evolved to mainly rely on vision for their survival. In chickens, there is behavioral evidence that the integration of visual and auditory stimuli is beneficial for signal detection [45]. However, even in the absence of visual stimuli, the chicken shows a significant accuracy in localizing frontal sound sources, which can be fundamental for its survival in critical conditions, for example to localize predators or conspecifics out of sight. However, there are still several open questions. For instance, given the fact that predators and possible threats may also come from above the animal, it would be interesting to investigate the localization accuracy along elevation and compare it with the acoustic cues available to accomplish this task.

## Supporting information

S1 File Methods and results of head movement analysis. (PDF)
