# Supplementary material for: Azimuthal sound localization in the chicken
Source: PLoS One. 2022 Nov 22;17(11):e0277190. doi: 10.1371/journal.pone.0277190 (PMC9681088; doi:10.1371/journal.pone.0277190)
Supplement: S1 File — (PDF) [file pone.0277190.s001.pdf]

Supporting information to: Azimuthal sound localization in the chicken

Gianmarco Maldarelli[1*], Uwe Firzlaff[1], Harald Luksch[1]

[1] Chair of Zoology, School of Life Sciences, Technical University of Munich, Freising-Weihenstephan, Germany

[*]Corresponding author

E-mail: gianmarco.maldarelli@tum.de

Supplementary Materials

In order to verify whether the chickens moved their head during localization of the long duration stimuli, for one chicken (Subject n.1) a 3D reconstruction of the head position and orientation was performed during closed-loop condition.

Setup

The mocap system (Flex 13, NaturalPoint Inc. DBA Optitrack, USA) consisted of 6 infrared (IR) cameras (refresh rate: 120 Hz) placed on the 4 walls of the behavioral chamber (2 on the back wall, 2 on the frontal wall, and 1 on each lateral wall), at a height between 80 and 90 cm above the floor. These cameras detected the IR-reflective markers placed on the chicken's head for a continuous recording of the 3D position and orientation during the task performance.

IR-Marker application

4 spherical IR-reflective markers were attached to the subject's head; two metal rods with thread endings were pierced through the comb, and one was pierced through the skin at the neck. The rods at the comb had one spherical marker on one side, and a nut on the opposite side; the rod at the neck had markers on both endings. To obtain an unambiguous pattern and hence a better detection by the cameras, an asymmetric arrangement of the markers was chosen. The whole procedure was performed under general anesthesia: an initial dose was injected (ketamine: 40 mg/kg; xylazine: 12 mg/kg), followed by maintained anesthesia with injections every half an hour (ketamine: 13 mg/kg; xylazine: 4 mg/kg). Moreover, a local analgesic lidocaine (Xylocain, AstraZeneca GmbH, Germany) was applied to the skin regions before the piercing operation. All protocols and procedures were in accordance with the institutional guidelines of the authorities of Upper Bavaria, Germany (permit no. ROB-55-2-2532-Vet_02-18-154).

Mocap data collection and analysis

During each trial the chicken performed the closed-loop condition task as before the application of the markers. The acquisition and representation of the 3D data of the head was accomplished online by the commercial software Motive:Tracker (Optitrack, USA). The 3D configuration of the markers was treated by the software as a rigid body. The center of the rigid body was the medial point along the interaural axis, and 3 axes were identified, orthogonal to each other: 1) the interaural axis; 2) the frontal axis, connecting the interaural axis to the beak tip; 3) a third axis orthogonal to the plane where the 2

other axes laid on. The position and orientation of the head in the following analysis refer to the distance and angle from the standard condition, respectively: the standard position is defined as the distance along the 3 axes of the rigid body center from the calibration point, placed close to the entrance of the chamber; the standard orientation corresponds to the head position when the animal is facing toward the central loudspeaker (LS) with a beak angle of ~45°. The standard orientation was recorded by the software when the animal was placed in the behavioral box, after the marker application and still under anesthesia. The head was held in the desired position using a head holder with ear bars covered with foam.

Thus, 6 parameters were recorded during each frame: the position of the head along the 3 axes (defined as the distance from the calibration point), and the angles along the 3 orthogonal planes (called yaw, on the horizontal plane; pitch, on the sagittal plane; and roll, on the frontal plane). Moreover, we defined 3 periods of interest: 1) the reference sound presentation; 2) the target sound presentation; 3) the response time, between the end of the target presentation and the pecking of the response key by the subject.

During the data analysis, the raw data were first pre-processed in 2 steps: discarding trials with big data loss, and data smoothing.

In some trials, the rigid body defined by the software was not continuously detected. In those cases, a continuous 'frozen' value in the position/rotation of the rigid body was recorded for a relative long time. If this data collection interruption was too long (>70 consecutive frames, corresponding to 0.6 s), the corresponding trial was discarded from further analysis.

Moreover, in some cases the markers were not continuously detected during all recordings due to particular positions/orientations of the head or temporary displacement of the markers. This resulted in artifacts especially in the reconstruction of the head orientation. In order to get rid of these artifacts, a smoothing was applied to the raw data by using the averaged mean (smoothing window length = 50 frames; MATLAB function 'movmean'). All data analysis was performed by custom-written scripts in MATLAB (MathWorks, USA).

Supplementary results

Mocap data from 310 trials were collected from subject 1. For each trial, the time series of the head position and orientation along each axis was extracted (Fig S1). Only a small percentage of trials were removed due to a relatively high data loss (39/310; 13%). Since the aim was to investigate the behavioral strategy used by the chicken during actual sound localization, in the following analysis we only included the trials with correct responses (192/271; 71%).

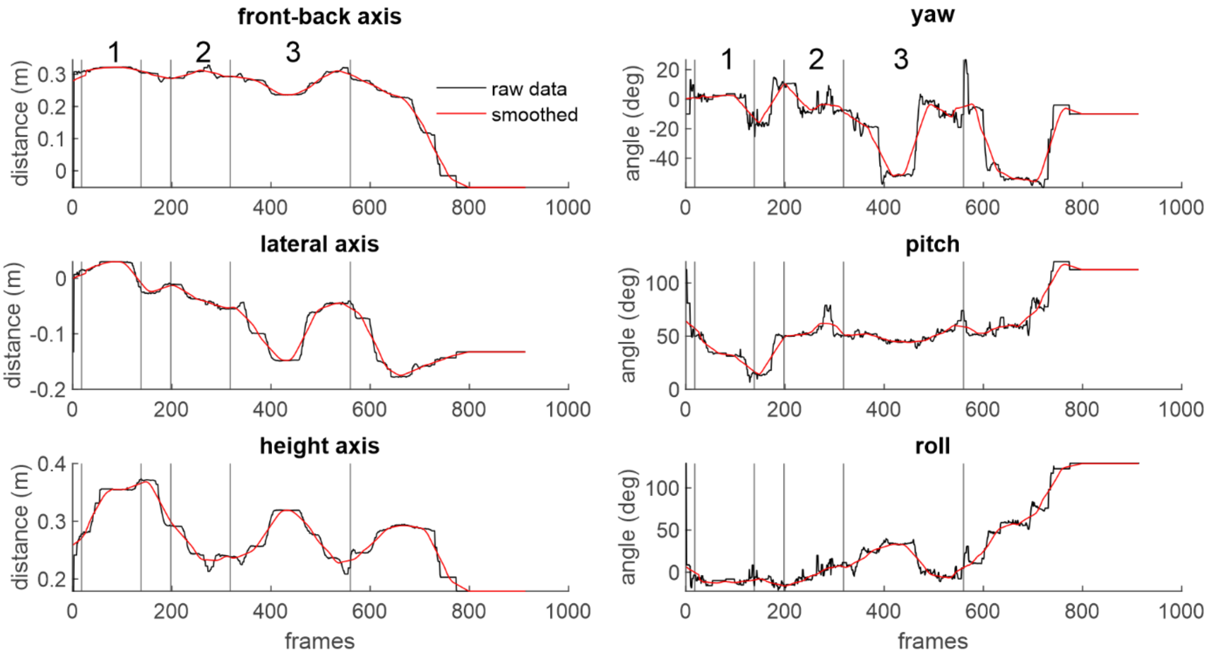

Fig S1. Time series of the head position and orientation. Example of raw and smoothed data of the head position track along the 3 axes and the orientation along the 3 planes. The periods labeled as 1, 2 and 3 refer to the reference stimulus, target stimulus presentation and response period, respectively.

Head position

From the smoothed data it was possible to 3D-reconstruct the position of the head during the reference and target sound presentation (Fig S2 A-B). In general, the chicken actively moved the head during both sound presentations. The average distance travelled was 13 ± 3 cm (mean ± S.D.) during reference sound and 13 ± 4 cm (mean ± S.D.) during the target sound. During the reference presentation, the head usually moved from the initiation key up to the center of the LS hoop, in front of the reference LS (Fig S2 A). However, after 0.5 s, during the target presentation, the head mainly moved toward two areas, which correspond to the lateral response keys (Fig S2 B). It is noteworthy that the period when the response was valid – thus, providing the reward if correct – was limited to the post-target period, when the lateral LEDs also turned on, as an additional visual cue. Any response during the target presentation

did not elicit reward (or punishment), but still the animal showed a tendency to at least approach the response key during the 1-s long target presentation. As expected, during the response period the chicken pressed one of the two response keys (Fig S2 C).

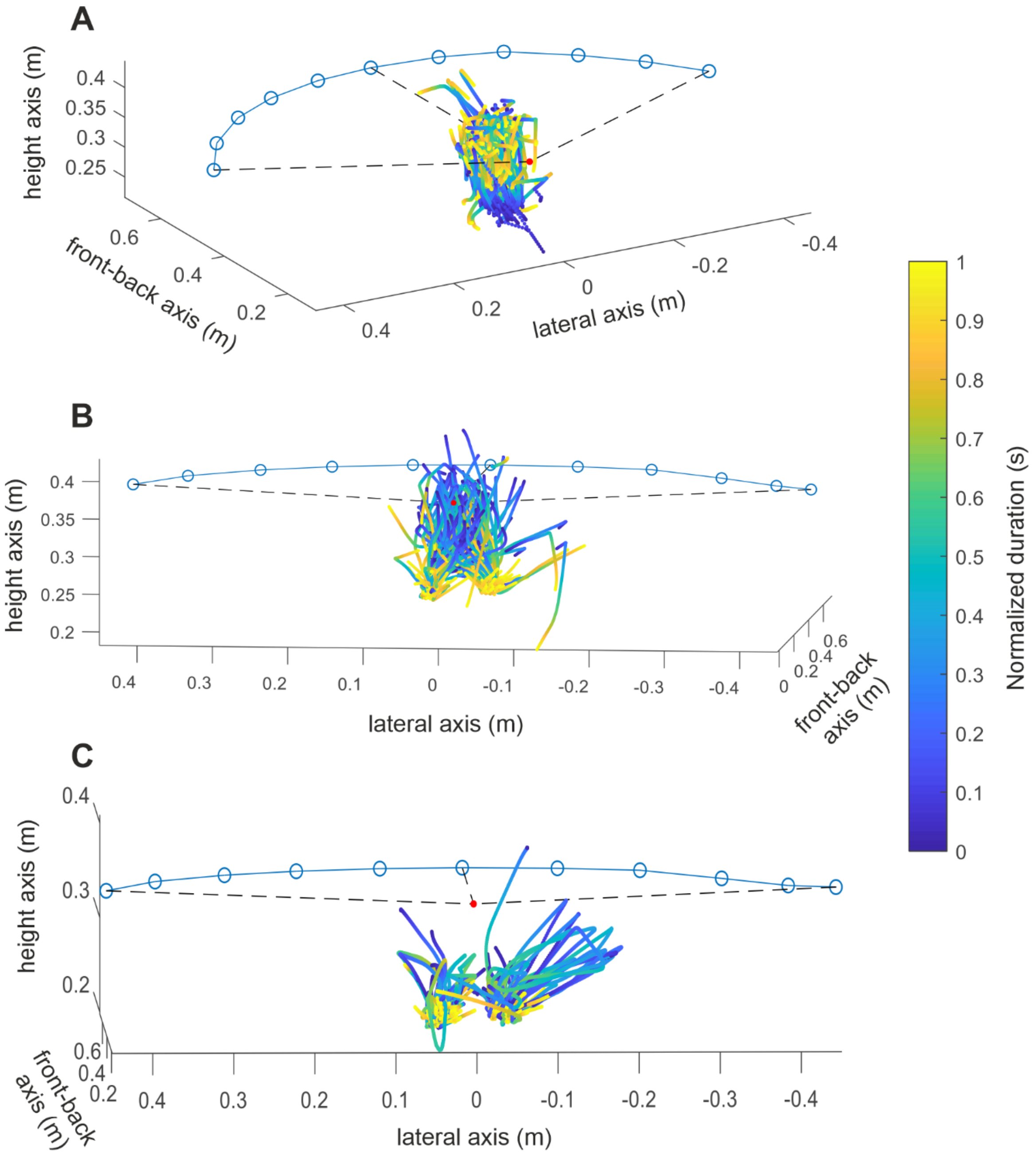

Fig S2. Head position during trial periods. Each line indicates the 3D head position trajectory for a single trial during A) the reference presentation, B) the target presentation and C) the response period. The color code indicates the time dimension within the period of interest. The blue circles represent the LSs, the red dot indicates the LS hoop center, connected to the most lateral and central reference loudspeakers by the dashed black lines.

Yaw and position in azimuth

After analyzing the head position alone, we investigated the combination of both position and orientation along the azimuthal plane, to additionally verify whether the subject simply oriented toward the response keys and not toward the sound sources. By considering both the position and orientation on the horizontal plane (yaw), we measured the intersection of the frontal axis with the LS hoop at each frame. Then, for each trial, we measured the median angle of intersection within each period of interest. These values were grouped by the period type in the histograms shown in Fig S3. During the reference sound the subject was mainly facing toward the central LS, whereas during the target sound the head had two preferred orientations, corresponding to the position of the 2 response keys, and with a biased orientation to the left. The pattern during the target sound well overlaps with the orientation during the post-stimuli response time, when the animal pecked one the lateral response keys.

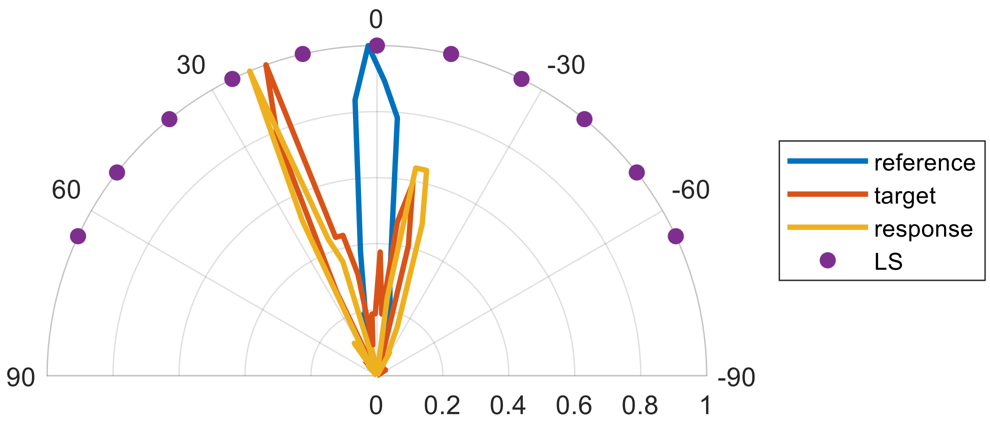

Fig S3. Normalized histograms of the frontal axis intersecting the LS hoop for each period. The bin width of the histograms is 3 degrees.

In conclusion, the chicken was actively moving and rotating the head during the presentation of long duration stimuli, even though it was not related to an active orientation reflex toward the stimulus, but rather as an anticipatory movement toward the response keys.

As mentioned in the Discussion, the change in the ITD/ILD reaching the eardrums as a consequence of the head movement might be one of the reasons of the increased localization accuracy in the closed-loop condition compared to open-loop condition.
